# Supplementary material for: Antibiotic Stewardship in Retail Pharmacies and the Access-Excess Challenge in China: A Policy Review
Source: Antibiotics (Basel). 2022 Jan 21;11(2):141. doi: 10.3390/antibiotics11020141 (PMC8868274; doi:10.3390/antibiotics11020141)
Supplement: Supplementary file 1 [file antibiotics-11-00141-s001.zip › antibiotics-1555465-supplementary.pdf]

antibiotics

MDPI

Review

Antibiotic Stewardship in Retail Pharmacies and the Access-Excess Challenge in China: A Policy Review

Tingting Zhang [1,*], Helen Lambert [1], Linhai Zhao [2], Rong Liu [2], Xingrong Shen [2], Debin Wang [2] and Christie Cabral [3]

Table S1. List of included articles

**Policy documents and reports**
1. China Drug Administration (CDA) 1999A. *Measures for the Supervision and Administration of Circulation of Pharmaceuticals (Elementary edition)* [Invalid]. Beijing: CDA.
2. CDA 1999B. *Measures for Separate Administration of Prescribed and Non-prescribed Medicines (Elementary edition)*. Beijing: CDA.
3. CDA 1999C. *Temporary Provisions for Prescribed and Non-prescribed Medicine Distribution.* Beijing: CDA.
4. CDA 1999D. *Notice on the introduction of list of first branch of national over the counter medicines (Western medicine and Chinese patent medicine) by Centre Drug Administration.* Beijing: CDA.
5. China Food and Drug Administration (CFDA) 2003A. *Notice on Strengthening Administration over the Sale of Antimicrobials in Retail Pharmacies to Promote Rational Use of Medicines.* Beijing: CFDA.
6. CFDA 2003B. *The Temporary Regulation Method on the Licensed Pharmacist Continuing Professional Development.* Beijing: CFDA.
7. CFDA 2004A. *Measures for the Administration of Pharmaceutical Trade License* [Revised]. Beijing: CFDA.
8. CFDA 2004B. *Notice on Publishing '2004-2005 Work Plan related to Separate Administration of Prescribed and Non-prescribed Medicines' by China Food and Drug Administration.* Beijing: CFDA.
9. CFDA 2007. *Measures for the Supervision and Administration of Circulation of Pharmaceuticals.* Beijing: CFDA.
10. CFDA 2016. *Good Supply Practice for Pharmaceutical Products (2016 Amendment).* Bei-

jing: CFDA.

11. CFDA 2017. *Decision of the China Food and Drug Administration to Amend Some Rules.* Beijing: CFDA.
12. Certification Center for Licensed Pharmacist of NMPA 2016. *Report on National Licensed Pharmacist Registration of January 2016.* Beijing: Certification Center for Licensed Pharmacist of NMPA [Online] http://www.cqlp.org/cqlpadminmanage/upfiles/201602/20160202162755581.pdf accessed 14/May/2021.
13. Ministry of Health (MoH)2010. *Notice on Issuing 'National Formulary (2010 edition)' by Ministry of Health.* Beijing: MoH.
14. MoH 2012A. *Administrative Regulations for the Clinical Use of Antibiotics.* Beijing: MoH.
15. MoH (Department of Medical Affairs Administration) 2011. *The 2011 Proposal of National Special Campaign for the Clinical Use of Antibiotics.* Beijing: MoH.
16. MoH (Department of Medical Affairs Administration) 2012B. *The 2012 Proposal of National Special Campaign for the Clinical Use of Antibiotics.* Beijing: MoH.
17. MoH (Department of Medical Affairs Administration) 2013. *The 2013 Proposal of National Special Campaign for the Clinical Use of Antibiotics.* Beijing: MoH.
18. MoH, National Traditional Chinese Medicine Administrative Bureau & Health Department of Ministry of Logistics 2004. *Notice on Implementing Principles for Clinical Use of Antibiotics.* Beijing: MoH, National Traditional Chinese Medicine Administrative Bureau, and Health Department of Ministry of Logistics
19. Ministry of Human Resources (MoHR) & CDA 1999. *Temporary Regulations on Licensed Pharmacist Qualification.* Beijing: MoHRSS & CDA.
20. National Health and Family Planning Commission (NHFPC) 2016. *The National Action Plan to Curb Bacterial Resistance (2016-2020).* Beijing: NHFPC.
21. State Council 2009. *Implementation Plan of Main Areas of Health System Reform in the Near Future (2009-2011).* Beijing: State Council.
22. State Council 2012. *Notice on Publishing National Medicine Safety twelve-five' Programme by State Council*. Beijing: State Council.
23. Chan CK, Ngok KL & Phillips D 2008. *Social Policy in China: Development and Wellbeing.* Bristol: Policy Press.
24. Mossialos E, Ge Y, Hu J et al. 2016. *Pharmaceutical Policy in China: Challenges and Op-*

portunities for Reform. Geneva: World Health Organisation.
25. WHO 2015B. *People's Republic of China Health System Review.* Manila: WHO Regional Office for the Western Pacific.
**Research studies**
26. Fang Y 2014. China should curb non-prescription use of antibiotics in the community. *BMJ* **348**:g4233.
27. Fang Y, Yang S, Zhou S et al. 2013. Community pharmacy practice in China: past, present and future. *International Journal of Clinical Pharmacy* **35**:520-528.
28. Li H & Sun H 2014. The historical evolution of China's drug regulatory system. *Value in Health* **17**: A30-A31.
29. Sun Q, Santoro MA, Meng Q et al. 2008. Pharmaceutical policy in China. *Health Affairs* **27**:1042-1050.
30. Xiao YH & Li LJ 2013. Legislation of clinical antibiotic use in China. *The Lancet Infectious Diseases* **13**:189-191.
31. Xiao YH & Li LJ 2015. The actions of China in containing antimicrobial resistance. *AMR Control*: 46-53.
32. Xiao YH, Zhang J, Zheng BW et al. 2013. Changes in Chinese policies to promote the rational use of antibiotics. *PLOS Medicine* **10**:e1001556.
33. Yu M, Zhu Y, Song X et al. 2013. Insights into residents' behaviour of antibiotic purchasing from medical sale of retail pharmacies in rural China. *Fudan University Journal of Medical Science* **40**:253-258.
34. Zhang Y 2011. The legislation of antibiotics. *Journal of Fangyuan* **23**:18-22.
